# Upscaling of thermoacoustic-Stirling duplex cryocoolers based on resonance tube coupling

**DOI:** 10.1016/j.isci.2025.113240

**Published:** 2025-07-30

**Authors:** Liping Wei, Haojie Sun, Hangyu Ma, Guoyao Yu, Shunmin Zhu, Wei Dai, Ercang Luo

**Affiliations:** 1 Key Laboratory of Cryogenic, Chinese Academy of Sciences, Beijing 100190, China; 2 University of Chinese Academy Sciences, Beijing 100049, China; 3 Institute of Optical Physics and Engineering Technology, Qilu Zhongke, Jinan 251000, China; 4 Department of Engineering, Durham University, DH1 3LE Durham, UK

**Keywords:** Electrochemical energy conversion, Thermoacoustics, Applied sciences

## Abstract

To address the critical need for energy-efficient and environmentally sustainable cooling technologies, the thermoacoustic-Stirling duplex cryocooler (TSDC) offers a promising solution due to its high efficiency and versatile applications. However, the limited cooling capacity of individual TSDCs poses a significant challenge for practical implementation, necessitating system upscaling. To enhance power density and performance, we optimized a resonance tube-coupled TSDC by incorporating a coiled resonance tube and developing an active displacer modeling approach. A prototype with optimized dimensions was constructed and tested, demonstrating a linear relationship between cooling power at 130 K and input heating power, with a maximum cooling capacity of 380 W achieved at an exergy efficiency of 6.36%. Compared to prior studies, which reported no cooling power at 110 K, this represents a substantial advancement in TSDC scalability. Comparative analysis of experimental and theoretical results suggests that asymmetric flow at the compression junction adversely affects efficiency, providing valuable insights for further optimization.

## Highlights

• Proposed active displacer method for optimizing thermoacoustic-Stirling duplex cryocooler
• A duplex cryocooler prototype with a coiled resonance tube modeled, constructed, and tested
• System achieves 380 W cooling capacity at 130 K, surpassing the best previous results
• An overall exergy efficiency of 6.36% with 15 kW heating power demonstrated

## Graphical abstract

**Figure:**
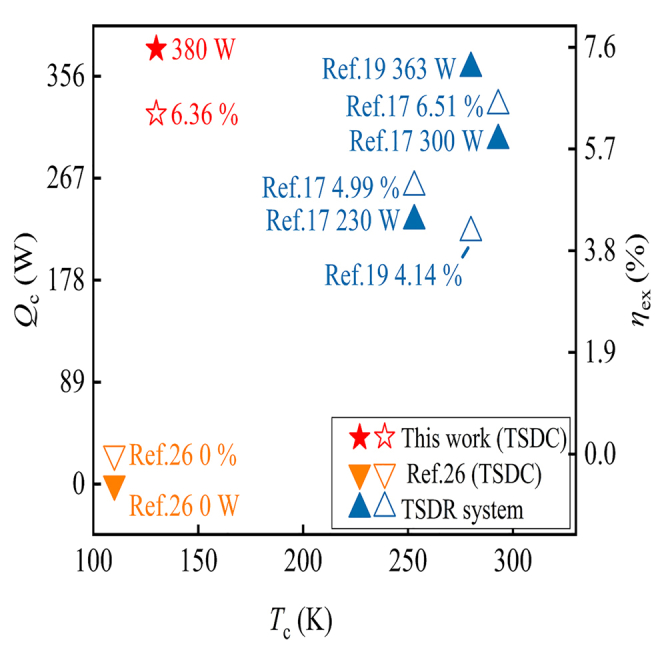

## Introduction

The escalating global energy crisis and rising levels of environmental pollution highlight the inherent limitations of conventional refrigeration technologies. In particular, systems that rely on chemical refrigerants—such as hydrofluorocarbons (HFCs), hydrofluoroolefins (HFOs), and hydrochlorofluorocarbons (HCFCs)—have been shown to significantly contribute to ozone layer depletion and the intensification of the greenhouse effect.^1^^,^^2^ In contrast, the Stirling cryocooler,^3^ which employs non-toxic, inert working fluids such as helium within the framework of the reversible Stirling cycle, offers notable advantages in both environmental sustainability and operational efficiency. This technology shows great potential across a wide range of applications, including low-temperature refrigeration,^4^^,^^5^ gas liquefaction,^6^ high-temperature superconducting system,^7^ and space exploration,^8^ among others.

In the context of Stirling cryocoolers, a duplex configuration enables the direct conversion of external heat into cooling power without the need for an intermediate energy conversion stage. A duplex Stirling cryocooler essentially consists of a Stirling engine unit that drives a Stirling cooler unit, both operating on the Stirling thermodynamic cycle.^9^ When heat is supplied to the heater of the engine unit from an external source, the Stirling engine generates acoustic power —a form of mechanical power— which is subsequently used to operate the Stirling cooler. The cooler then transfers heat from the cold source to the ambient sink. The method of coupling between the Stirling engine and cooler units is critical to the overall system performance. Generally, two main coupling approaches are employed: mechanical coupling, which utilizes a physical piston mechanism, and acoustic coupling, which employs a resonance tube to transmit power acoustically.

The mechanical coupling-based duplex Stirling system was first proposed by Dr William Beale in 1969,^10^ predating the development of acoustic coupling-based configurations by several decades. The earliest implementation of this concept involved two Stirling machines sharing a common power piston and a sealed pressure enclosure.^9^ Recent research has focused on optimizing TSDC performance through innovative design and operational enhancements. For instance, studies on motion mechanisms in Stirling systems have provided valuable insights. Specifically, Erol et al. experimentally compared three motion mechanisms —rhombic drive,^11^ crank-slider, and bell-crank^12^— in a beta-type Stirling engine, identifying friction losses in moving components as the primary factor influencing performance differences. The rhombic drive mechanism outperformed the crank-slider and bell-crank, achieving a maximum engine power of 215.48 W with helium gas at 4 bar pressure.^13^ Additionally, investigations into the internal working principles of duplex Stirling systems^14^^,^^15^ have underscored the critical role of coupling methods in determining overall system efficiency. Based on the dynamic piston coupling approach, Hu et al.^16^ proposed a novel system design and performed simulations using dynamic equilibrium equations, predicting an efficiency of 30.6% at a cooling capacity of 1.9 kW. However, this result has not yet been experimentally validated. Similarly, a cooling and power cogeneration system^17^ employing an oscillating linear alternator for coupling, achieved an experimental cooling power of approximately 230 W at 253 K. Nevertheless, the system’s performance was found to be highly sensitive to the operational parameters of the linear alternator. To further improve refrigeration performance, a thermoacoustic-Stirling duplex refrigerator (TSDR) system^18^^,^^19^ coupled with a thermal buffer tube was developed, delivering 363 W of cooling power at 280 K. Despite these advancements, all of the aforementioned systems remain constrained to operate within the room temperature range.

As a matter of fact, the essential coupling mechanism for the aforementioned TSDC system is mechanical, which inevitably compromises system reliability and increases sensitivity to mechanical tolerances. In contrast, purely acoustic coupling eliminates issues related to the degrees of freedom (DOF) of coupling pistons, offering a more robust and maintenance-free alternative. A two-stage pulse tube cryocooler driven by a traveling-wave thermoacoustic–Stirling engine has demonstrated cooling temperatures of 66.6 K and 18.7 K at the first and second stages, respectively.^20^^,^^21^^,^^22^ Expanding on this approach, a looped-cascade thermoacoustic-driven cryocooler^23^ system that achieved a peak cooling capacity of 670 W at 130 K was developed, with an overall system efficiency of 8.3%. However, the use of a multi-unit loop architecture results in a dispersed system layout and inconsistent energy distribution across the various components,^24^ thereby limiting its practicality in compact or integrated applications.

Given the advantages of acoustic resonance tubes in enhancing reliability and compactness,^25^ researchers have investigated their integration into TSDC systems. Li et al.^26^ proposed employing a resonance tube as the coupling element in a TSDC configuration. In this design, the resonance tube replaces the conventional mechanical piston, resulting in a simulation model without active acoustic components. To achieve system matching, a virtual piston was introduced, whose displacement was adjusted to maintain dynamic balance. While experimental results demonstrated a no-load cooling temperature of 110 K, the system failed to achieve an effective cooling capacity, primarily due to suboptimal coupling between the engine and cryocooler units. Additionally, the system’s design was hindered by its large physical footprint, as it incorporated a 6-meter-long straight resonance tube, significantly reducing its power density. The virtual piston method (VPM) employed in earlier studies assumed a uniform flow field and considered only the dynamic equilibrium at the piston ends. However, such simplifications are inadequate for acoustically coupled resonance tubes, which exhibit substantial spatial flow variations. To overcome these limitations, the present work proposes a novel joint analysis methodology —referred to as the active displacer method (ADM)— which integrates fluid dynamic control equations with dynamic balance equations to optimize resonance tube coupling in TSDC systems. Furthermore, a coiled resonance tube is introduced to replace the previously used straight tube, significantly increasing system power density. Through numerical simulations and experimental validation, this study aims to provide new insights into improving the performance and practical viability of TSDC systems.

## Results and discussion

### System configuration and numerical study

**Figure 1:**
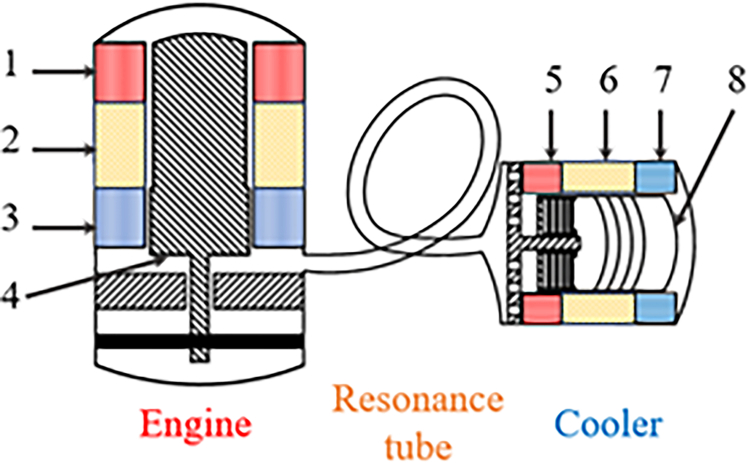
Schematic diagram of the TSDC system: 1. HHX_E_, 2. REG_E_, 3. AHX_E_, 4. DIS_E_, 5. AHX_C_, 6. REG_C_, 7. CHX_C_, 8. DIS_C_ The system includes an engine unit, a resonance tube unit and a cooler unit.

Figure 1 shows a schematic diagram of the TSDC system. As illustrated, the TSDC system investigated in this study primarily comprises three main components, i.e., an engine unit, a cooler unit, and a resonance tube unit. The engine unit includes a hot heat exchanger (HHX_E_, where the subscript “E” denotes the engine unit), an engine regenerator (REG_E_), an ambient heat exchanger (AHX_E_), and an engine displacer (DIS_E_). Correspondingly, the cooler unit consists of an ambient heat exchanger (AHX_C_, with the subscript “C” indicating the cooler unit), a cooler regenerator (REG_C_), a cold heat exchanger (CHX_C_), and a cooler displacer (DIS_C_). The engine and cooler units are interconnected by two segments of regularly coiled resonance tubes.^27^ The dimensional specifications of the TSDC components are listed in Table 1. In comparison with the previously employed 6-meter-long straight resonance tube assembly,^26^ the use of coiled tubes reduces the horizontal length to approximately 0.4 m, thereby significantly improving the system’s power density.

**Table 1:** Structural parameters of the TSDC system

Units	Components	Length (mm)	Diameter (mm)	Spring Stiffness (kN/m)	Porosity (%)
Engine	HHX_E_	67	180	/	/
	REG_E_	60	180	/	90
	AHX_E_	62	180	/	/
	DIS_E_	120	/	426	/
Cooler	AHX_C_	30	102	/	/
	REG_C_	59	102	/	76
	CHX_C_	35	102	/	/
	DIS_C_	80	/	102	/
Resonance tube	Tube _1_	4000	32	/	/
	Tube _2_	1700	45	/	/

**Figure 2:**
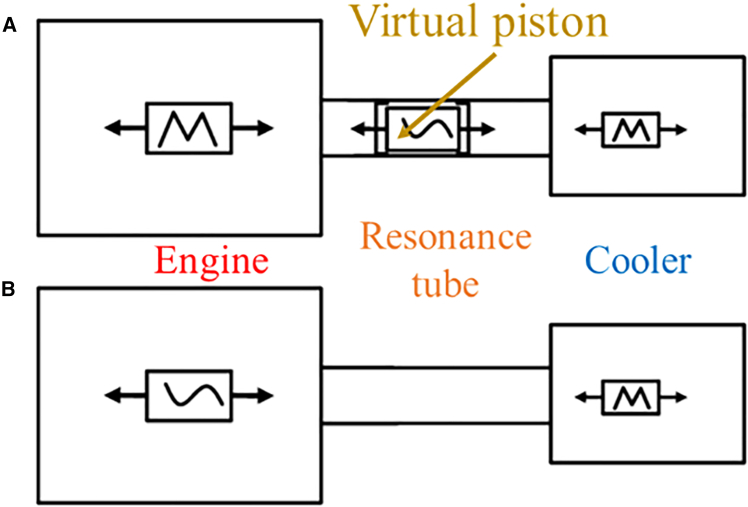
Coupling structure diagram of the TSDC system (A) The virtual piston method. (B) The active displacer method. The symbol “M” on the displacer pistons represents the displacer is modeled as a reciprocator in the SAGE software, and the tilde means the displacer piston or the resonance tube is modeled as a constrained piston.

Although the stand-alone acoustic performance of the coiled resonance tube has been validated,^27^ its integration into a TSDC remains a significant challenge, thereby necessitating s comprehensive, system-level simulation approach. Before continuing with detailed calculations, it is essential to address the issue of coupling criteria between subsystems. In our previous work,^28^ utilizing the commercially available software SAGE, we proposed the VPM. This method introduces a virtual piston that ensures dynamic equilibrium at the piston ends while neglecting spatial variations in the flow field (see Figure 2A). In contrast, the ADM models the engine displacer as a constrained piston (Figure 2B), and involves a coupled analysis of both fluid dynamics and dynamic equilibrium equations. The resonance tube is discretized into multiple nodes according to its geometric distribution, and the governing equations are sequentially solved at each node. The solution mechanism ensures that the net force acting on every node is zero, i.e., *F* = 0, where *F* denotes resultant force, thus capturing the coupled dynamic behavior more accurately.

The performance of the system is evaluated using several efficiency metrics: resonance tube efficiency (*η*_t_), engine efficiency (*η*_e_), cooler efficiency (*η*_c_), and the overall exergy efficiency (*η*_ex_). The definitions of these efficiency parameters are provided in Equations 1, 2, 3, and 4.

(Equation 1) $$\eta_{t}=W_{\text{ac}}/W_{\text{ae}}$$

(Equation 2) $$\eta_{e}=W_{\text{ae}}/Q_{h}$$

(Equation 3) $$\eta_{c}=Q_{c}/W_{\text{ac}}$$

(Equation 4) $$\eta_{\text{ex}}=\frac{Q_{c}\left(T_{A}/T_{C}-1\right)}{Q_{h}\left(1-T_{A}/T_{H}\right)}$$

In these expressions, *W*_ac_ and *W*_ae_ denote the acoustic power at the cooler and engine side, respectively; *Q*_h_ and *Q*_c_ represent the heating and cooling powers, respectively; *T*_A_, *T*_C_, and *T*_H_ correspond to the ambient, cooling, and heating temperatures, respectively.

Table 2 summarizes the simulation results obtained using the VPM and the ADM. Notably, the VPM predicts an overall exergy efficiency (*η*_ex_) that is approximately 14.90% higher than that predicted by the ADM. This discrepancy is primarily attributed to turbulent flow phenomena within the engine’s compression chamber, where multiple fluid streams converge. The VPM, by assuming a uniform flow at the resonance tube inlet, neglects these turbulent effects, resulting in a slight overestimation of *η*_ex_. Additionally, the resonance tube length optimized by the VPM exceeds that obtained by the ADM by 200 mm. This increased length contributes to greater viscous dissipation, leading to a higher onset temperature, which in turn may prevent the system from initiating oscillation.^28^

**Table 2:** Simulation results optimized using the VPM and the ADM

	Resonance tube diameter (mm)	Resonance tube length (mm)	*Q*_h_ (W)	*Q*_c_ @110K (W)	*η*_e_ (%)	*η*_t_ (%)	*η*_c_ (%)	*η*_ex_ (%)
VPM	32	6510	12.85	1172	72.62	61.73	54.51	24.44
ADM	32	6300	12.33	978.7	73.86	55.6	51.79	21.27

### Experimental results and discussion

**Figure 3:**
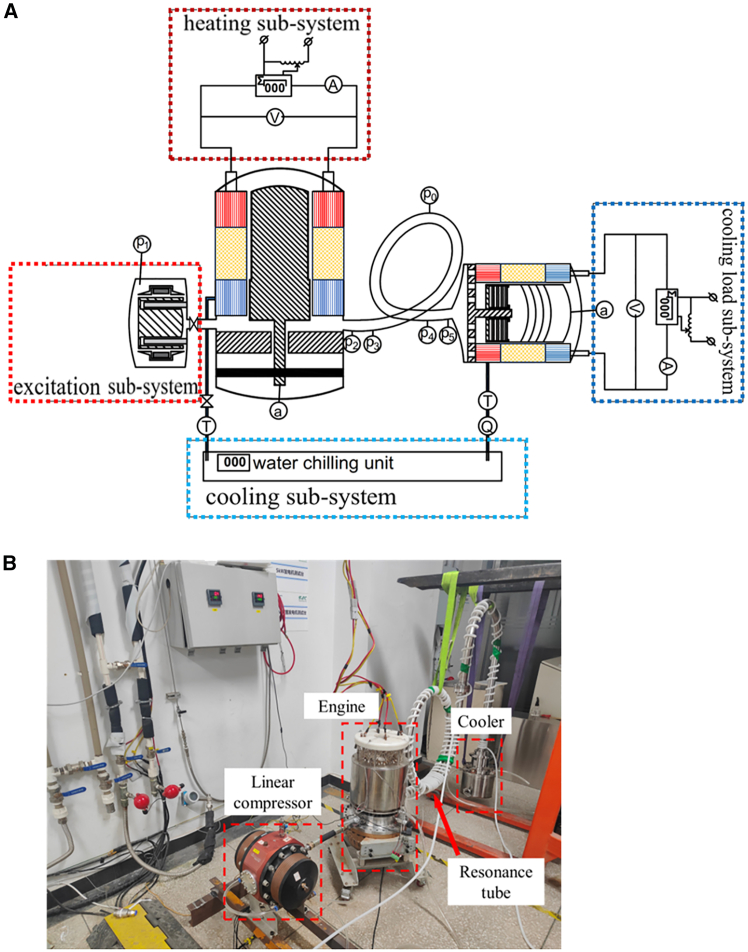
Structural schematic and experimental photograph of the TSDC system (A) Schematic diagram of the test system. The symbols “P”, “T”, “a”, and “Q” denote the pressure sensor, thermometer, accelerometer, and flowmeter, respectively. The subscript numbers following “P” indicate the sequence of pressure sensors. The sub-systems include heating, excitation, cooling, and cooling load sub-systems. (B) Photograph of the test rig of the TSDC system.

Based on the simulation results, an experimental test rig was constructed to validate the system’s performance. Figure 3A shows the schematic diagram of the test setup, while Figure 3B presents a photograph of the assembled test rig. As depicted in Figure 3A, the test system comprises four core subsystems: a heating subsystem employing electric heating, an excitation subsystem featuring a linear compressor and a shut-off ball valve, a cooling subsystem, and a cooling load subsystem. It should be noted that a linear compressor and a shut-off ball valve are included to facilitate system startup. Once the system initiates operation, the shut-off ball valve is closed, and the linear compressor is deactivated. As depicted in Figure 3B, the free-piston Stirling engine unit is mounted on a vibration-damping base, while the cooler unit is suspended from a support frame to minimize vibration transmission. The experimental Stirling engine retains identical design characteristics to the simulated model, with detailed parameters listed in Table 3. The engine configuration includes the following core components: a hot heat exchanger (HHX_E_, where the subscript “E” denotes the engine unit), an engine regenerator (REG_E_), an ambient heat exchanger (AHX_E_), an engine displacer (DIS_E_), an expansion space (EXP_E_), and a compression space (COM_E_). In accordance with the experimental design, two coiled resonance tube segments are employed as coupling elements to enhance system power density. One segment measures 4000 mm in total length with a diameter of 32 mm, while the other measures 1700 mm in length with a diameter of 45 mm.

**Table 3:** Characteristics of the experimental Stirling engine

Components	Length (mm)	Diameter (mm)	Volume (mm^3^)	Spring Stiffness (kN/m)	Porosity (%)
HHX_E_	67	180	/	/	/
REG_E_	60	180	/	/	90
AHX_E_	62	180	/	/	/
DIS_E_	120	/	/	426	/
EXP_E_	/	/	320	/	/
COM_E_	/	/	426	/	/

**Figure 4:**
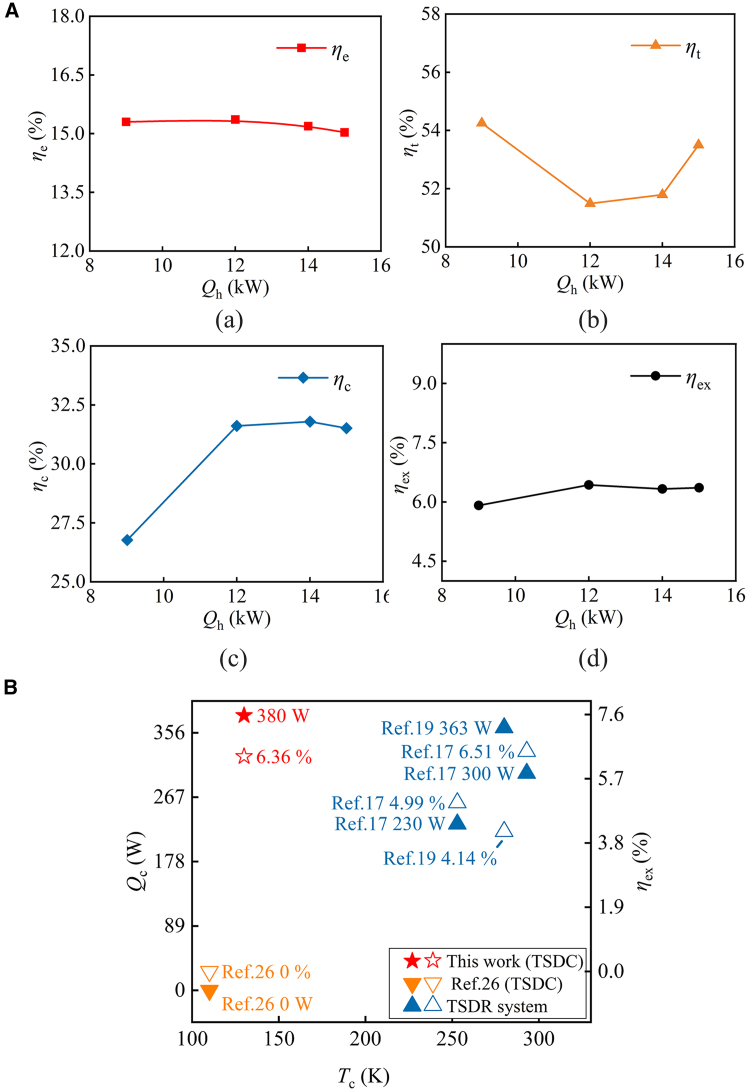
Efficiency response to heating power and comparative performance of the TSDC system (A) Influence of heating power (*Q*_h_) on system efficiencies including: (a) engine efficiency (*η*_e_), (b) resonance tube efficiency (*η*_t_), (c) cooler efficiency (*η*_c_), and (d) exergy efficiency of the overall system (*η*_ex_). (B) The cooling power (*Q*_c_), cooling temperature (*T*_C_), and overall exergy efficiency (*η*_ex_) of the TSDC system compared with other thermoacoustic-Stirling duplex systems presented in published papers.

Experimental investigations were conducted on the optimized TSDC system using both the VPM and the ADM. The resonance tube dimensions optimized via ADM successfully enabled self-sustained oscillations, whereas those derived from the VPM failed to initiate onset. The experimental performance of the ADM-optimized TSDC system is summarized in Table 4. As shown, the system achieves a maximum cooling power of 380 W at 130 K under a heating power of 15 kW, corresponding to an overall exergy efficiency of 6.36%. Further analysis indicates that the system attains its peak overall exergy efficiency of 6.43% at a reduced heating power of 12 kW, albeit with a slight decline in cooler-side thermal efficiency. Figure 4A illustrates the influence of heating power (*Q*_h_) on system efficiencies. It can be observed that the engine efficiency remains relatively constant at approximately 15% as the heating power increases from 9 kW to 15 kW, which is consistently lower than the cooler efficiency across the measured range. Figure 4B compares the performance of the present thermoacoustic-Stirling duplex refrigeration (TSDR) system with other duplex systems reported in the literature. As illustrated in Figure 4B, duplex systems employing mechanical coupling, such as those described by Refs. ^17^ and ^19^, whose cooling temperatures are limited to within the room temperature range. In contrast, a TSDC system utilizing acoustic coupling achieved a cooling temperature of 110 K, albeit with no reported cooling power.^26^ In this study, our TSDC system demonstrated a cooling capacity of 380 W at 130 K, marking the first validated substantial cooling capacity in this cryogenic regime. This performance corresponds to an overall exergy efficiency of 6.36%, representing a significant advancement over previously reported thermoacoustic-Stirling duplex refrigeration systems.

**Table 4:** Experimental results of the TSDC system

*Q*_h_ (kW)	*Q*_c_ @130K (W)	Acoustic power at engine side (W)	Acoustic power at cooler side (W)	Engine displacer displacement (mm)	Cooler displacer displacement (mm)
9	200	1377	747	6.71	2.77
12	300	1843	949	7.68	3.08
14	350	2126	1101	8.36	3.29
15	380	2254	1206	8.72	3.58

Table 5 compares system efficiencies obtained from simulations and experiments, highlighting notable discrepancies that warrant detailed evaluation. Specifically, the experimental efficiency of the resonance tube exhibits a positive deviation of 19.02% relative to the calculated value. This deviation is primarily attributed to overestimations in the model concerning surface roughness, local flow losses, and friction effects in the coiled resonance tube. As a result of this overestimation, the experimental efficiency of the cooler is 10.46% lower than that predicted by simulation.

**Table 5:** Comparison of system performance between simulation and experiment

	*η*_e_ (%)	*η*_t_ (%)	*η*_c_ (%)	*η*_ex_ (%)
Calculation	47.01	44.95	35.19	15.25
Experiment	15.03	53.50	31.51	6.36

Initially, the substantial discrepancy of 58.30% in the overall system exergy efficiency was perplexing. However, insights from a combined cooling and power (CCP) experimental setup—where the auxiliary linear compressor operates as a linear alternator to generate electricity (illustrated in Figure S3)—shed light on the issue (details of the CCP experiment are provided in the Supplementary Information and Table S1). Under the TSDC operating mode, the unilateral bifurcation configuration induces an asymmetric flow field, which severely disrupts the flow within the compression chamber and leads to an imbalance in energy distribution within the engine unit.

Superimposed direct current (DC) flow on alternating current (AC) flow is a common phenomenon in this field, though it varies in configuration and magnitude. As demonstrated by Gedeon,^29^ even a small DC flow component (as little as 0.5% of the AC amplitude) in closed-loop thermoacoustic systems can induce parasitic thermal gradients. These gradients distort the temperature profiles within the regenerator and pulse tube, ultimately degrading enthalpy transport. In our system, the proven flow asymmetry disrupts the energy balance in the compression chamber. This observation is consistent with the findings of Yang,^30^ who reported that the direction of local DC flow significantly influences the temperature distribution in the hot heat exchanger and affects the overall cooling capacity. Particularly relevant to the pronounced flow asymmetry observed here, Spoor and Corey^31^ showed that in large-scale (approximately 200 W @ 80 K) coaxial cold finger pulse tube cryocoolers, such asymmetries—including substantial circumferential temperature gradients in the regenerator and nonlinear cooling capacity responses—become increasingly prominent at higher power levels. Their work also demonstrated that adding flow straighteners or designing for geometrical symmetry effectively mitigates these nonuniformities, thereby enhancing system performance.

Taken together, these discrepancies indicate that the primary limitation on overall system performance stems from low engine efficiency caused by flow asymmetry. This underscores the need for further investigation into the underlying mechanisms and potential design improvements, with the goal of significantly enhancing the system’s exergy efficiency.

### Conclusion

• This study presents a coiled resonance tube-coupled thermoacoustic-Stirling duplex cryocooler (TSDC) system for LNG temperature operation and introduces a novel lossless active displacer analysis method for holistic simulation.
• Experimental results reveal that resonance tube dimensions optimized via the virtual piston method (VPM) fail to initiate oscillation, whereas the system designed using the active displacer method (ADM) achieves a cooling power of 380 W at 130 K with 15 kW heating power, yielding 6.36% overall exergy efficiency.
• This performance surpasses prior studies in both no-load temperature and cooling capacity, marking a significant advancement and providing preliminary validation of the simulation model.
• Moreover, the findings underscore the importance of mitigating flow asymmetry to enhance system performance, which will be the primary focus of future research.

### Limitations of the study

This study is subject to several limitations. First, although mitigating flow asymmetry is emphasized as crucial for enhancing system performance, the distortion of the acoustic field and its impact on thermoacoustic conversion were not thoroughly investigated. Future research should incorporate systematic multi-dimensional computational modeling and experiments to investigate this. Second, for quantifying acoustic power within the resonance tube, the double-sensor method was employed, using dynamic pressure sensors at both the inlet and outlet of the resonance tube. While this approach yields necessary data, more accurate methodologies should be explored in subsequent studies. Finally, the experimental results were primarily compared with previous reports, which have no cooling power at a lower temperature, i.e., 110 K.^26^ However, our measurements are currently limited to 130 K. The lack of directly comparable data at 110 K prevents a fair assessment under identical conditions. Therefore, comprehensive experimental characterization across the relevant temperature range is essential in future investigations.

## Resource availability

### Lead contact

Further information and requests for resources and reagents should be directed to the Lead Contact, Shunmin Zhu (shunmin.zhu@durham.ac.uk).

### Materials availability

This study did not generate new unique reagents.

### Data and code availability

• Data: The data that support the findings of this study available from the corresponding author upon reasonable request.
• Code: This study utilized established analytical methods with no custom code generated. All software implementations followed vendor-recommended protocols as detailed in the key resources table section.
• Other items: No additional unique resources requiring specialized access procedures were generated in this study.

## Acknowledgments

This research was financially supported by the National Natural Science Foundation of China (Grant No. 51876214 and 52306031). This research was also supported by the European Union’s Marie Skłodowska-Curie Actions Individual Fellowship (No. MSCA-IF-101026323).

## Author contributions

Methodology, H.S., H.M., and G.Y.; formal analysis: L.W., H.S., and H.M.; writing – original draft, L.W. and H.S.; writing – review and editing, G.Y., H.M., and S.Z.; supervision, G.Y., S.Z., W.D., and E.L.

## Declaration of interests

The authors declare no competing interests.

## STAR★Methods

### Key resources table

**Table:** 

REAGENT or RESOURCE	SOURCE	IDENTIFIER
**Deposited data**		

Data generated for this paper	This paper	N/A

**Software and algorithms**		

SAGE	Gedeon Associates	Sage software
Microsoft Excel	Microsoft Corporation	https://www.microsoft.com/enca/microsoft-365/excel

### Method details

#### Simulation model and subject details

##### Details of the virtual piston method (VPM)

Given the inherent advantages of acoustic resonance tubes, such as improved reliability and compactness,^25^ researchers have investigated their integration into thermoacoustic-Stirling duplex cryocooler (TSDC) systems. Li et al.^26^ proposed employing a resonance tube as the coupling element in a TSDC system. In this configuration, the resonance tube replaces the traditional mechanical piston, resulting in the absence of active acoustic elements in the simulation model. To address this limitation, a virtual piston was introduced to facilitate system matching by adjusting its displacement.

In our previous work,^28^ we formalized this concept as the virtual piston method (VPM) using the commercially available simulation software SAGE. Figure S1 presents the SAGE program chart for a resonance tube-coupled free-piston Stirling cooler modeled with VPM, where the virtual piston and resonance tube are highlighted in red boxes. The VPM assumes a uniform flow field and considers only the dynamic equilibrium at the piston ends, neglecting spatial variations in the acoustic flow (as shown in Figure 2A). However, this simplification becomes inadequate in the context of acoustically coupled resonance tubes, which inherently exhibit significant spatial variation in both pressure and velocity fields.

##### Details of the active displacer method (ADM)

The Virtual Piston Method (VPM) employed in previous studies assumes a uniform flow field and considers only the dynamic equilibrium at both ends of the piston. However, this simplification is inadequate for acoustically coupled resonance tubes, which exhibit significant spatial variations in pressure and velocity fields.

To overcome these limitations, the present study introduces a novel joint analysis approach, referred to as the Active Displacer Method (ADM), which integrates fluid dynamic control equations with dynamic balance equations to optimize the resonance tube coupling in the TSDC system. In this method, the engine displacer is modeled as a constrained piston (as illustrated in Figure 2B), and a coupled analysis of the flow field and mechanical dynamics is performed. The resonance tube is discretized into multiple nodes according to its geometric and distribution characteristics. The governing equations are then solved sequentially at each node, ensuring that the resultant force on every node is zero, i.e., *F* = 0, where *F* represents the resultant force. This condition effectively defines the solution mechanism and enables accurate prediction of the coupled system behavior. Figure S2 shows the SAGE program chart of the resonance tube-coupled free-piston Stirling cooler using ADM, with the resonance tube component highlighted in a red box.

#### Experimental system and subject details

##### Configuration details of the TSDC experimental system

Based on the simulation results, an experimental test rig for the TSDC system was constructed. Figure 3A illustrates the schematic layout of the test system, while Figure 3B presents a photograph of the assembled apparatus. As shown in Figure 3A, the system comprises four primary subsystems: a heating subsystem, an excitation subsystem, a cooling subsystem, and a cooling load subsystem. Detailed descriptions of each subsystem are provided below:

(1) Heating subsystem: The heating subsystem supplies thermal energy to the free-piston Stirling engine. To enable accurate performance measurement, electric heating was employed in this experiment. The subsystem includes heating cartridges evenly distributed on the hot heat exchanger, an AC power supply, a voltmeter, an ammeter, and a power meter. The heating power can be adjusted by regulating the supply voltage to the heating cartridges.
(2) Excitation subsystem: The excitation subsystem consists of a linear compressor and a shut-off ball valve. During the start-up phase, the gas bearings supporting the moving components are not yet operational, and the displacer requires an external pressure disturbance to overcome static friction and initiate motion. To address this, the linear compressor is externally connected to the compression chamber of the TSDC. During the onset process, transient pressure oscillations generated by the compressor piston help initiate system operation. Once stable self-sustained oscillations are achieved, the shut-off ball valve is closed, and the linear compressor is turned off.
(3) Cooling subsystem: The cooling subsystem comprises platinum resistance thermometers, a turbine flowmeter, and a water chiller connected in series. To ensure adequate cooling water flow, the ambient heat exchangers of both the engine and the cooler are connected in parallel to the water chiller. The chiller provides a rated cooling capacity of 9.09 kW, with its outlet water temperature maintained at 20°C during operation.
(4) Cooling load subsystem: The cooling load subsystem is composed primarily of a DC power supply and heating rods. The DC power supply energizes the rods, and the generated heat serves as a simulated thermal load. During testing, the cold heat exchanger of the cooler was maintained under vacuum conditions, effectively eliminating heat leakage to the environment. As a result, the heat input from the heating rods is assumed to equal the cooling capacity delivered by the cooler.

### Quantification and statistical analysis

All numerical simulations were performed using Sage software, with implementation details documented in the method details section and results validation presented in results and discussion. Primary data organization, descriptive statistics, and result tabulation were conducted in Microsoft Excel (Microsoft 365), with no inferential statistical tests (e.g., t-tests, ANOVA) applied beyond descriptive computations. All numerical values (simulation outputs and experimental data) are truncated to two decimal places unless otherwise specified.
