# Supplementary material for: Upscaling of thermoacoustic-Stirling duplex cryocoolers based on resonance tube coupling
Source: iScience. 2025 Jul 30;28(9):113240. doi: 10.1016/j.isci.2025.113240 (PMC12362697; doi:10.1016/j.isci.2025.113240)
Supplement: Document S1. Figures S1–S3, Tables S1, and Data S1 [file mmc1.pdf]

iScience, Volume *28*

Supplemental information

Upscaling of thermoacoustic-Stirling duplex cryocoolers based on resonance tube coupling

Liping Wei, Haojie Sun, Hangyu Ma, Guoyao Yu, Shunmin Zhu, Wei Dai, and Ercang Luo

Supplemental Figures and Legends

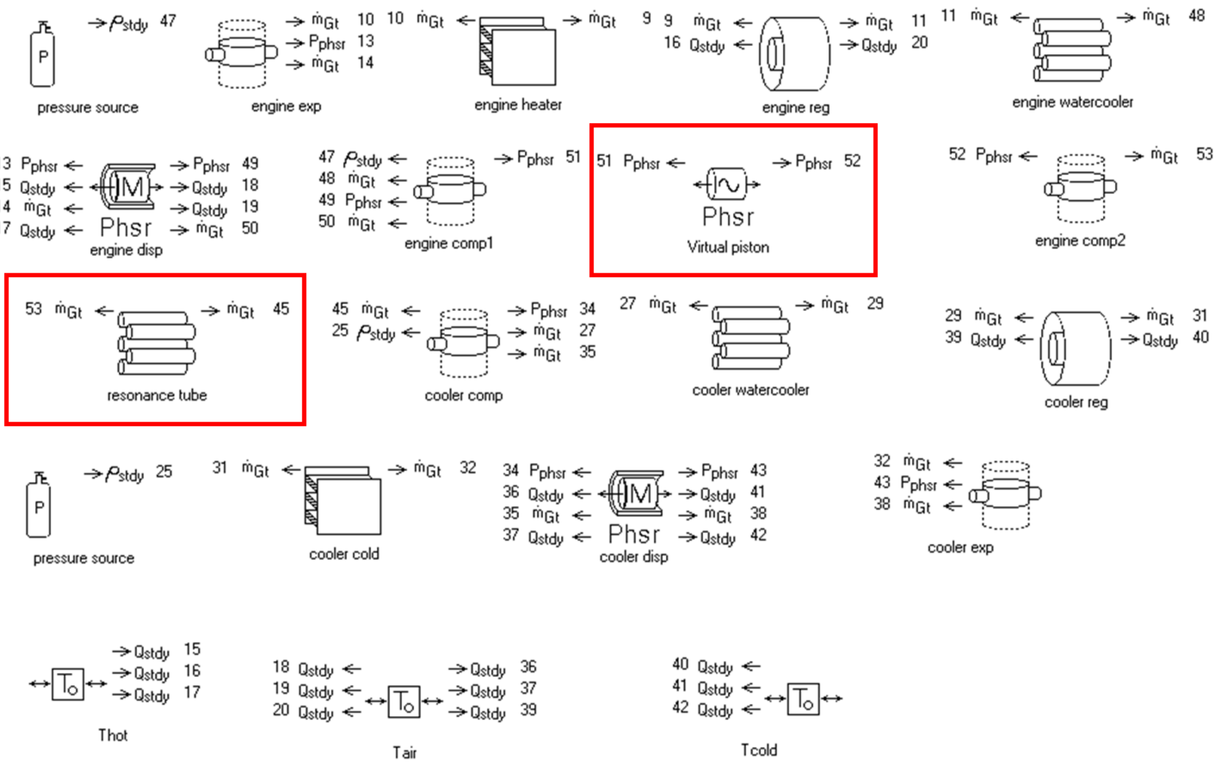

Figure S1. Program chart of resonance tube coupled free-piston Stirling cooler using the virtual piston method. Related to STAR METHODS, "METHOD DETAILS" subsection.

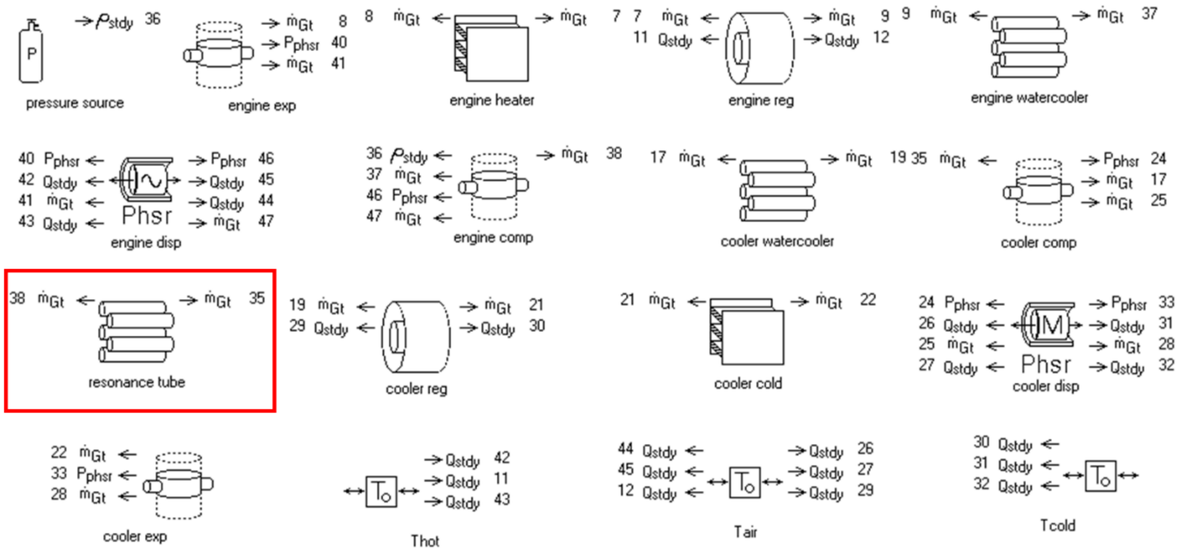

Figure S2. Program chart of resonance tube coupled free-piston Stirling cooler using the active displacer method. Related to STAR METHODS, "METHOD DETAILS" subsection.

A

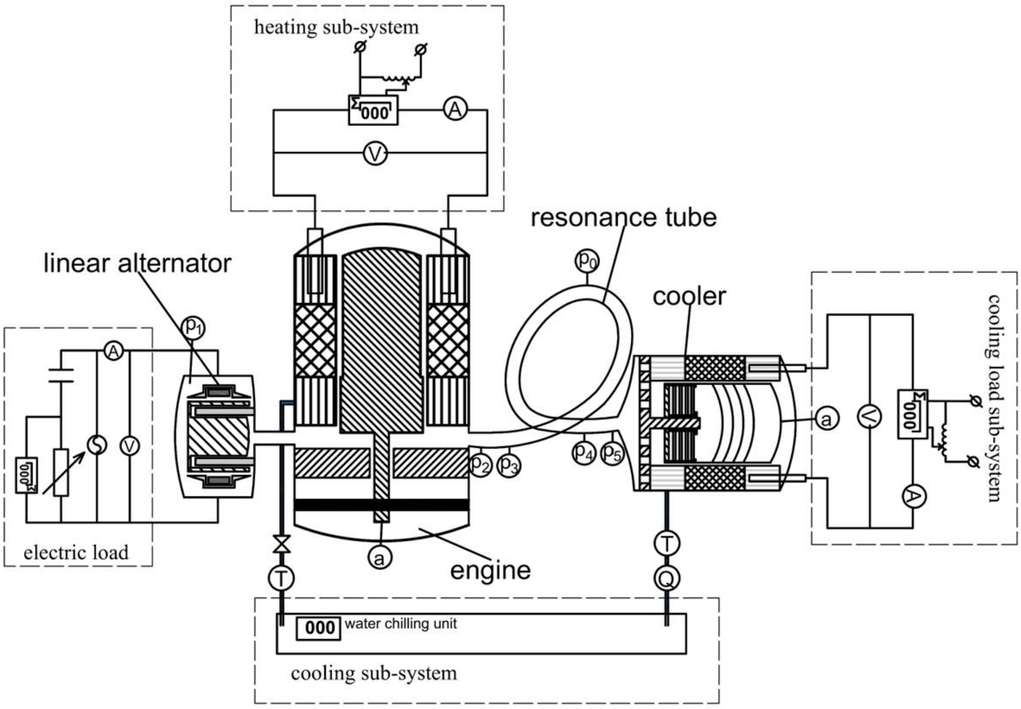

B

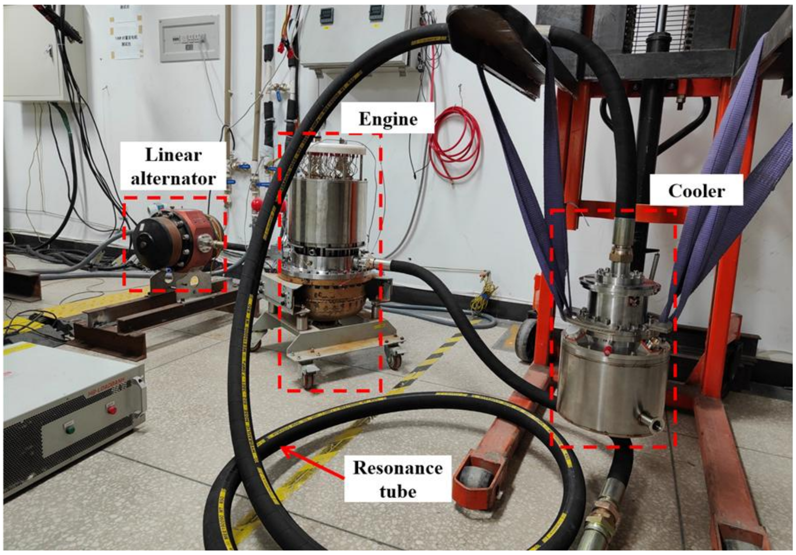

Figure S3. Schematic configuration and experimental implementation of the duplex Stirling cryocooler-based combined cooling and power system. (A) Schematic diagram of the system. and (B) Photograph of the physical prototype. Related to RESULTS AND DISCUSSION, "Experimental results and discussion" subsection.

Supplemental Tables and Legends

Table S1. Experimental performance of the duplex Stirling cryocooler-based combined cooling and power system under varying input heating power. Related to RESULTS AND DISCUSSION, "Experimental results and discussion" subsection.

Q_h (kW)	Q_c @130K (W)	Acoustic power on the engine side (W)	Acoustic power on the cooler side (W)	Engine displacer displacement (mm)	Cooler displacer displacement (mm)	Piston displacement (mm)	W_e (W)	Transmission efficiency of resonance tube (%)	η_t (%)	η_{ex} (%)
9	190	1215	787	8.6	2.7	10	554	64.77	4.36	14.08
11	250	1498	946	9.3	2.91	10.5	690	63.15	4.66	14.50
12	295	1684	1060	9.86	3.04	10.6	864	62.95	5.01	16.23
13	330	1884	1186	10.33	3.14	10.7	1030	62.95	5.11	17.30
14	350	2018	1258	10.66	3.21	10.9	1087	62.34	5.01	16.90
15	370	2156	1327	1094	3.29	11	1152	61.55	4.92	16.64

Data S1. Combined cooling and power experiment results and discussion.

This work demonstrated a duplex Stirling cryocooler-based combined cooling and power system. An experimental setup was constructed and tested. Figure S3A portrays the layout the resonance tube-coupled duplex Stirling cryocooler, including a free piston Stirling engine unit, a free piston Stirling cooler unit, an acoustic resonance tube unit and a linear alternator unit. Figure S3B is a photograph of the system. The linear alternator was integrated with the duplex Stirling cryocooler and functioned as an acoustic exciter before the onset of the self-sustained oscillation. Once the system onsets, the linear alternator will run as a generator to convert part of the acoustic power produced by the engine unit into electric power. Besides, the use of the linear alternator during the operation of the duplex Stirling cryocooler enhanced the synergic effect.

During the experiment, the linear alternator maintains an acoustic-to-electric efficiency of around 80% when subjected to a load resistance ranging from 139 Ω to 73 Ω. It could be deduced with caution that the linear alternator is weakly coupled in the system. Table S1 lists the experimental results under steady state with varying input heating power, indicating that a symmetric layout of the linear alternator and resonance tube around the engine unit's compression space demonstrates superior performance. Based on these observations, it was found that under the

thermoacoustic-Stirling duplex cryocoolers mode, the asymmetric flow field resulting from the single-sided inlet configuration overwhelmingly disturbs the flow in the compression chamber and leads to an imbalance in energy flow within the engine unit. In detail, the experiment results are reported in Ref. 1.

Supplemental Reference

1. Yu, G.Y., Ma, H.Y., Sun, H., and Dai, W. (2024) A Duplex Stirling Cryocooler with a Flexible Acoustic Resonator. In ICC23 Program of Papers, (International Cryocooler Conference), Session 4.5.
